# Ectopic Intrauterine Device in the Bladder of a Pregnant Woman

**DOI:** 10.1155/2010/181032

**Published:** 2010-08-02

**Authors:** Zehra Kurdoglu, Kadir Ceylan, Mertihan Kurdoglu, Ayse Guler, Hanim Guler Sahin

**Affiliations:** ^1^Department of Obstetrics and Gynecology, Faculty of Medicine, Yuzuncu Yil University, 65300 Van, Turkey; ^2^Department of Urology, Yuzuncu Yil University, 65300 Van, Turkey

## Abstract

*Background*. Uterine perforation and transvesical migration of an intrauterine device are rare complications. *Case*. A 28-year-old woman who had an intrauterine device was admitted to our outpatient clinic with complaints of amenorrhea lasting 5 weeks and pelvic pain lasting a year. Transvaginal ultrasonography revealed embedding of the intrauterine device in the bladder. The misplaced device was removed by laparotomy. *Conclusion*. The followup of intrauterine device localization with transvaginal ultrasonography is essential for early detection of possible serious complications.

## 1. Introduction

A major but infrequent complication of intrauterine device (IUD) insertion is perforation. The incidence of uterine perforation is 1–3 in 1000 applications [1]. However, migration of the IUD into an adjacent organ is rarely seen. 

In this report, we present a case of ectopic intrauterine device in the bladder diagnosed in a pregnant woman.

## 2. Case Report

**Figure 1:**
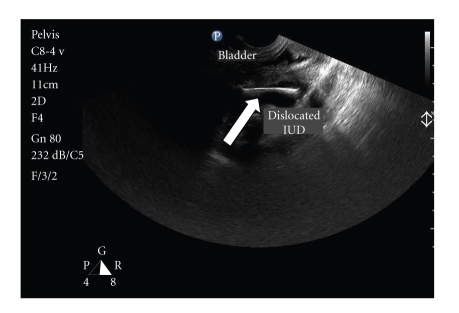
Transvaginal sonography showed a misplaced intrauterine device (arrow) that seemed to be located in the uterovesical space and embedded in the left posterior wall of the bladder.

 A 28-year-old woman, gravida 4 parity 3, was admitted to our outpatient clinic with complaints of secondary amenorrhea lasting 5 weeks and lower abdominal pain lasting a year. Physical examination findings and vital signs were within normal limits. The medical history of the patient revealed that a copper-T-380-A intrauterine device (IUD) had been inserted into the uterine cavity after her last vaginal delivery. The IUD strings were not visible on gynecologic examination. On transvaginal ultrasonography, the IUD appeared to be in the uterovesical space and embedded in the left posterior wall of the bladder (Figure 1). Additionally, a five-week gestational sac was determined in the uterine cavity. 

**Figure 2:**
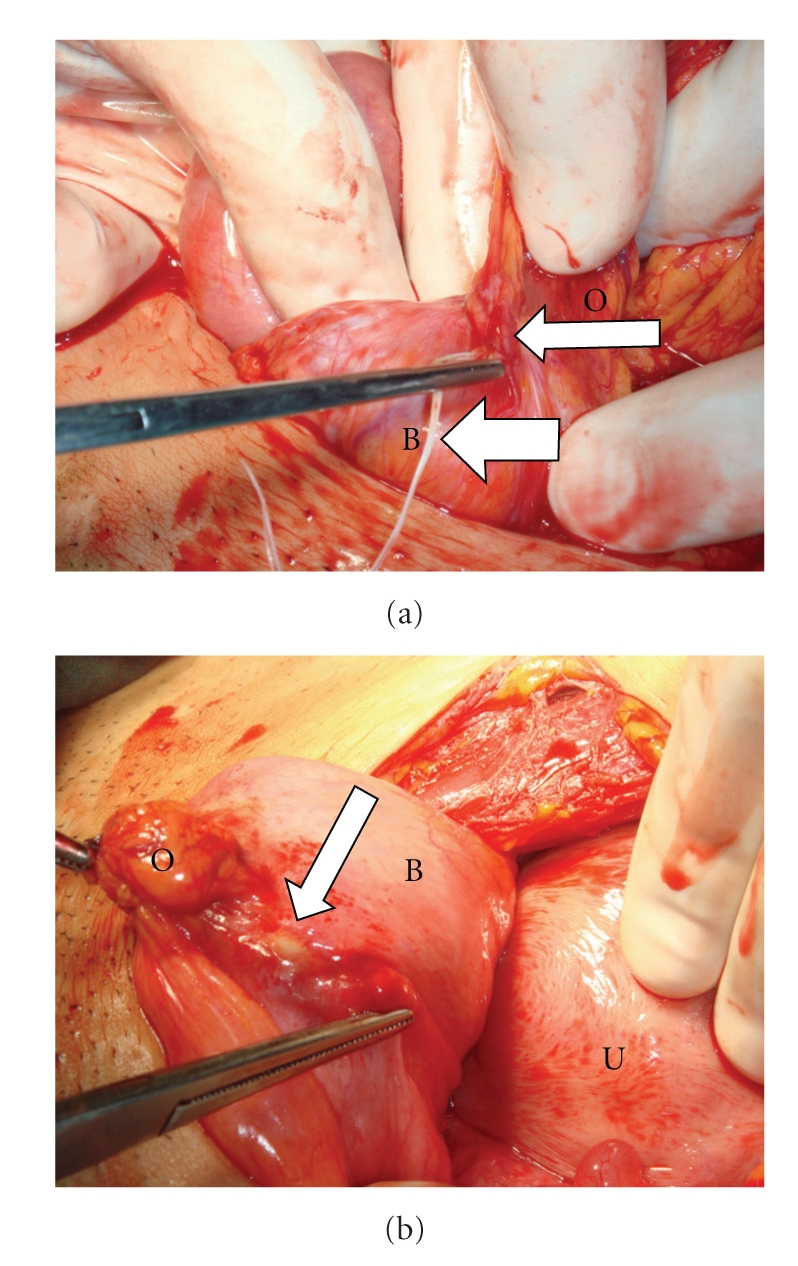
(a) Anterior view of the bladder. The thick arrow shows the string and the thin arrow shows the horizontal limb of the intrauterine device. Adhesions between the bladder (B) and omentum (O) are observed. (b) Posterior view of the bladder. The arrow shows the vertical limb penetrating the posterior wall of the bladder (B). Uterus (U) and omentum (O) are also shown.

The pregnancy was terminated upon the patient's request and then a laparotomy was performed. On exploration, dense adhesions between the bladder and omentum were observed. After dissection, the strings and horizontal limb of a copper-T-380-A IUD perforating the serosal layer of the bladder were observed, with the vertical limb penetrating the posterior wall of the bladder (Figure 2). 

While dissecting the IUD, pus drained spontaneously around the horizontal limb. The IUD was removed from the bladder through an incision. After the incision had been sutured, a catheter was placed in the bladder and the bladder was filled with fluid. After ensuring that no fluid leaked, a Foley catheter was left inside for 2 days postoperatively. Cefazolin sodium (1000 mg IV every 12 hours) and metronidazole (500 mg IV every 8 hours) were administered during her stay in hospital. The patient was discharged without any complication on postoperative day 2 with antibiotic therapy composed of cefuroxime axetil (500 mg PO twice a day) and metronidazole (500 mg PO twice a day).

## 3. Discussion

The IUD is one of the most popular methods of birth control in Turkey but it may be associated with some rare but serious complications such as uterine perforation and migration into adjacent organs [2–4]. The rate of uterine perforations is 1–3 per 1000 insertions [5]. If the strings of the IUD are not seen in the gynecologic examination, the risk of perforation and penetration into the adjacent organs like the bladder should be kept in mind. Ultrasonography is a useful tool when looking for a missing IUD [6]. While some patients experience hematuria, lower abdominal pain, and irritative urinary symptoms, others may be asymptomatic [7]. In our case, only lower abdominal pain and amenorrhea were present. With the help of transvaginal ultrasonography, we were able to easily detect the missing IUD, which had become embedded in the bladder wall.

Although calculus formation was evident in most of the presented cases in which an IUD had migrated into the bladder [2, 3, 8], it was not observed in our case. Instead, pus was observed around the IUD. 

It seems likely that the uterus was perforated in our case at the time of IUD insertion and caused the pain lasting 1 year. However, migration is also a possibility. Since removal of the migrated IUD due to its serious complications even in asymptomatic patients is recommended [9–11], we removed it.

In conclusion, the followup of IUD's with ultrasonography immediately after insertion and periodically thereafter may be helpful to avoid serious complications and undesired pregnancies.
